# Clinical Complexity in Native Vertebral Osteomyelitis: Exploring the Role of Rurality—A Retrospective Cohort Study

**DOI:** 10.3390/jcm15218455

**Published:** 2026-10-30

**Authors:** David Briz, Kimberly Rodríguez, Carmen da Casa, Samuel Gil, Guillermo García, Javier Pérez, Juan F. Blanco

**Affiliations:** 1 Facultad de Ciencias de la Salud, Universidad Pontificia de Salamanca, Calle Compañía, 5, 37002 Salamanca, Spain; 2 Facultad de Medicina, Universidad de Salamanca, 37007 Salamanca, Spain; 3 Servicio de Cirugía Ortopédica y Traumatología, Hospital Universitario de Salamanca, 37007 Salamanca, Spain; 4 Instituto de Investigación Biomédica de Salamanca (IBSAL), 37007 Salamanca, Spain

**Keywords:** native vertebral osteomyelitis, spondylodiscitis, rurality, rural health, multimorbidity, complications

## Abstract

**Introduction**: Native vertebral osteomyelitis (NVO) is an uncommon infection whose nonspecific presentation frequently delays diagnosis and treatment. Rurality is a potentially relevant but under-researched factor. This study aimed to identify factors independently associated with NVO-related complications at admission, with particular emphasis on rurality, and to describe the cohort’s clinical, etiological, and therapeutic profile. **Material and Methods**: We conducted an observational, retrospective cohort study of 67 consecutive adults with NVO admitted to the University Hospital of Salamanca between 2018 and 2023. Rurality was defined by primary healthcare zone of residence; nursing home residents formed a separate group. The primary outcome was the presence of at least one NVO-related complication at admission. A multivariable logistic regression model including age, sex, rurality, and multimorbidity was fitted, followed by two sensitivity analyses. **Results**: NVO-related complications occurred in 56.7% of patients and were more frequent among rural than non-rural patients (73.1% vs. 46.3%; *p* = 0.031). In the multivariable analysis, rurality (aOR = 4.88; 95% CI, 1.44–16.47; *p* = 0.011) and multimorbidity (aOR = 3.42; 95% CI, 1.00–11.65; *p* = 0.049) were independently associated with complications. The association between rurality and NVO-related complications remained consistent across both sensitivity analyses, although its magnitude was imprecise. **Conclusions**: Rurality may represent a relevant factor in the clinical course of NVO. Further investigation is required.

## 1. Introduction

Vertebral osteomyelitis, or spondylodiscitis, is an inflammatory condition caused by infection of the spinal column with pathogenic microorganisms. Depending on its mechanism of onset, it can be classified into postoperative osteomyelitis, the most common form, and native vertebral osteomyelitis (NVO). Although relatively uncommon, with a population incidence rate of approximately 3 to 5 cases per 100,000 inhabitants, NVO represents the third most frequent cause of osteomyelitis in individuals over the age of 50 [1]. Its incidence is currently increasing, likely linked to the rise in invasive medical procedures (e.g., catheterizations) and the growing population with substantial comorbidities. Clinically, NVO tends to present with nonspecific signs and symptoms, contributing to delayed diagnosis and treatment [2]. The most common symptom is back pain, present in approximately 86% of patients, although it is a sensitive but nonspecific finding among individuals over 60. Fever is reported in 60% of cases, while neurological deficits, radiculopathy, urinary retention, paralysis, or sensory loss are less frequently observed [3].

**Figure 1:**
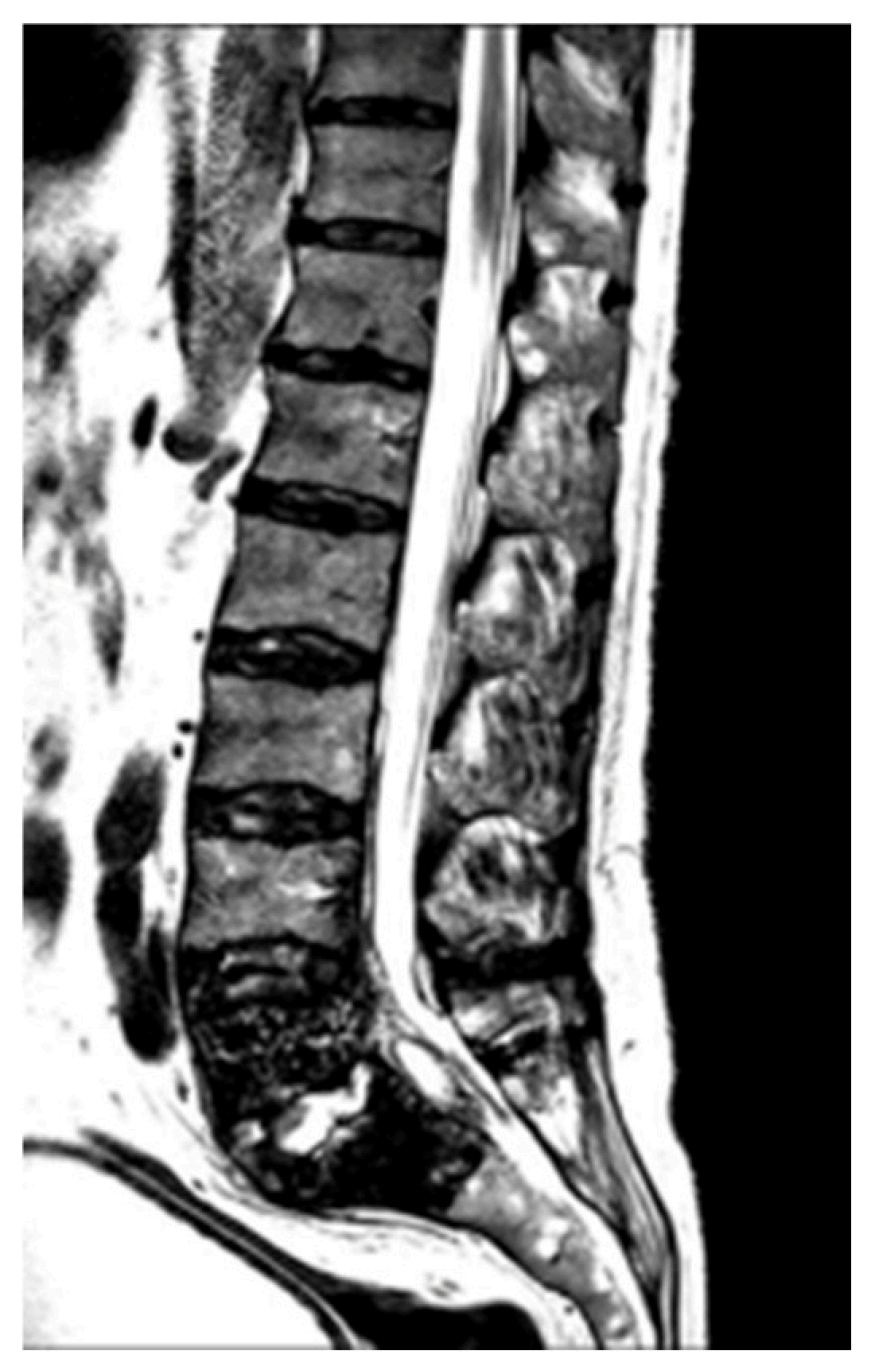
Native vertebral osteomyelitis with epidural abscess requiring surgical drainage. Sagittal view on MRI.

In NVO, microorganisms reach the vertebral column from a primary or secondary infection source, commonly via hematogenous spread or contiguous extension. The primary focus is identified in only half of patients and is usually located in the urinary tract, skin, or soft tissues [4]. In adults, intervertebral discs are avascular, which facilitates infection originating in the adjacent vertebral endplates, subsequently affecting the disc space. Septic emboli cause ischemia and infarction, leading to structural destruction, spinal instability, deformity, and risk of spinal cord compression. If left untreated, the infection may spread to adjacent tissues, forming paravertebral or epidural abscesses (Figure 1) with the potential for severe complications such as paraplegia or meningitis. The thoracic spine is affected in 52% of cases and the lumbar spine in 43% [5].

According to etiology, NVO may be classified as pyogenic, granulomatous, parasitic, or fungal. *S. aureus* is the most common cause worldwide [6], accounting for approximately half of all cases, followed by other pyogenic infections. Parasitic and fungal causes are rare [4]. Among pyogenic pathogens, *Staphylococcus aureus* is the most frequently isolated, followed by *Escherichia coli*, *Proteus* spp., *Klebsiella* spp., *Enterobacter spp.*, and *Pseudomonas aeruginosa*, which tend to involve the lumbar region [7]. In 21–34% of cases, no specific pathogen is identified [5].

Diagnosis of NVO is based on compatible clinical findings, supported by imaging and laboratory tests. Magnetic resonance imaging (MRI) is considered the gold standard for the diagnosis of NVO, while computed tomography (CT) may be useful when MRI is contraindicated. Laboratory tests, including acute-phase reactants and C-reactive protein (CRP) and erythrocyte sedimentation rate (ESR), are commonly used, with reported sensitivity ranging from 94% to 100% [3]. Blood cultures may be useful when clinical suspicion is established; however, they are frequently negative, and CT-guided biopsy is often required to identify the causative pathogen.

Regarding treatment, antibiotic therapy remains the cornerstone of management, although controversy persists regarding therapeutic strategy, including duration and antibiotic spectrum. A significant number of patients will require surgical intervention as part of their management [8].

Given the nonspecific nature of clinical presentation, delays in diagnosis are frequent and have been associated with poor outcomes [6,9]. In this line, rurality deserves particular attention as a sociodemographic characteristic that may aggregate determinants of healthcare and diagnostic access, which may differ from those in urban settings [10,11]. Previous studies in other conditions considering the role of rurality have highlighted barriers to care access that may lead to delays and interruptions [12], while others have underlined the limitations imposed by distance from the reference center [13]. Burdorf et al. (2022) addressed this problem in terms of accessibility to diagnostic imaging. The clinical course of NVO is highly heterogeneous and reflects the interaction between multiple sociodemographic, host-, pathogen-, and diagnosis-related factors [4,5,6,7,8,14,15,16]. Consequently, patients with apparently similar disease presentations may experience markedly different clinical trajectories and outcomes ranging from a complete recovery to major disease-related complications, even death. From this perspective, NVO may be understood as a clinically complex disease, in which the occurrence of disease-related complications represents one of the earliest clinically relevant manifestations of disease progression and may serve as a primary outcome of disease progression reflecting the underlying complexity of its clinical course [6]. In this study, we consider rurality as one of these potential sociodemographic factors that may condition the primary outcomes. Rurality is understood as a sociodemographic proxy variable that may aggregate some of the underlying individual-level factors described above.

Despite the published studies on NVO, the available evidence remains heterogeneous and is largely based on retrospective series with limited sample sizes. Furthermore, the role of certain sociodemographic and clinical factors in disease progression and complications of the disease remains incompletely understood, and few studies have comparatively analyzed their possible influence on clinical outcomes. Particularly, rurality is an interesting and potentially relevant risk factor that remains under-researched. On the one hand, evidence supporting the impact of rurality on clinical outcomes in other musculoskeletal diseases already exists [17,18]. On the other hand, although the evidence specifically addressing rurality in spinal infections is sparse and heterogeneous, several clues suggest that rurality may also have a relevant impact on clinical outcomes in this context. Descriptive studies by Guo et al. and Ribeira et al. found high proportions of rural patients in their cohorts, reaching 63% and 71%, respectively [19,20]. In another study, Baran et al. observed an association between rurality and brucellar spondylodiscitis [21], whereas Kim and Kim identified rurality as a factor associated with the development of postoperative spinal infection [22]. Furthermore, Pomponio et al. found that patients transferred between hospitals, who were more frequently from rural areas, had higher mortality than non-transferred patients [23]. Finally, and most importantly for the purposes of our study, Yilboudo et al. reported that 90% of patients presenting with neurological manifestations requiring surgical intervention came from rural areas [24].

Taken together, these clues do not demonstrate that rurality is independently associated with complications, but they provide the rationale for investigating its potential role in NVO. To the best of our knowledge, no previous study has specifically investigated rurality in NVO or, more specifically, evaluated its association with NVO-related complications.

We therefore hypothesized that rurality, understood as a sociodemographic proxy variable, would be associated with the presence of NVO-related complications.

The aim of this study was to identify factors independently associated with the presence of disease-related complications in patients with NVO, with particular emphasis on the potential role of rurality.

As a secondary objective, we sought to describe the clinical, etiological, and therapeutic profile of our cohort of patients with NVO treated at a tertiary university hospital.

## 2. Materials and Methods

### 2.1. Study Design, Setting and Population

After ethical approval from the Reference Ethics Committee (Ref. PI 2024 04 1582-TFG), we conducted an observational, retrospective cohort study including patients diagnosed with Native Vertebral Osteomyelitis (NVO), also referred to as spondylodiscitis, who were admitted to the University Hospital of Salamanca from 2018 to 2023. Patients were identified using ICD-10 codes ICD-M46.2, ICD-M46.3 and ICD-M46.4. This study is reported in accordance with the STROBE recommendations (Supplementary material Table S1: STROBE Checklist).

Patients were identified from the hospital administrative database, managed by the Clinical Documentation Department of the University Hospital Complex of Salamanca (Complejo Asistencial Universitario de Salamanca, CAUSA, Salamanca, Spain), after a formal request, using the ICD-10 classification system with diagnostic codes. Once the dataset was obtained, to minimize potential inconsistencies, a direct clinical verification procedure was used instead of relying solely on automatic mappings between coding systems. Patients were classified as having Native Vertebral Osteomyelitis by cross-checking the administrative code against the clinical diagnosis recorded in the medical record by the physicians.

**Figure 2:**
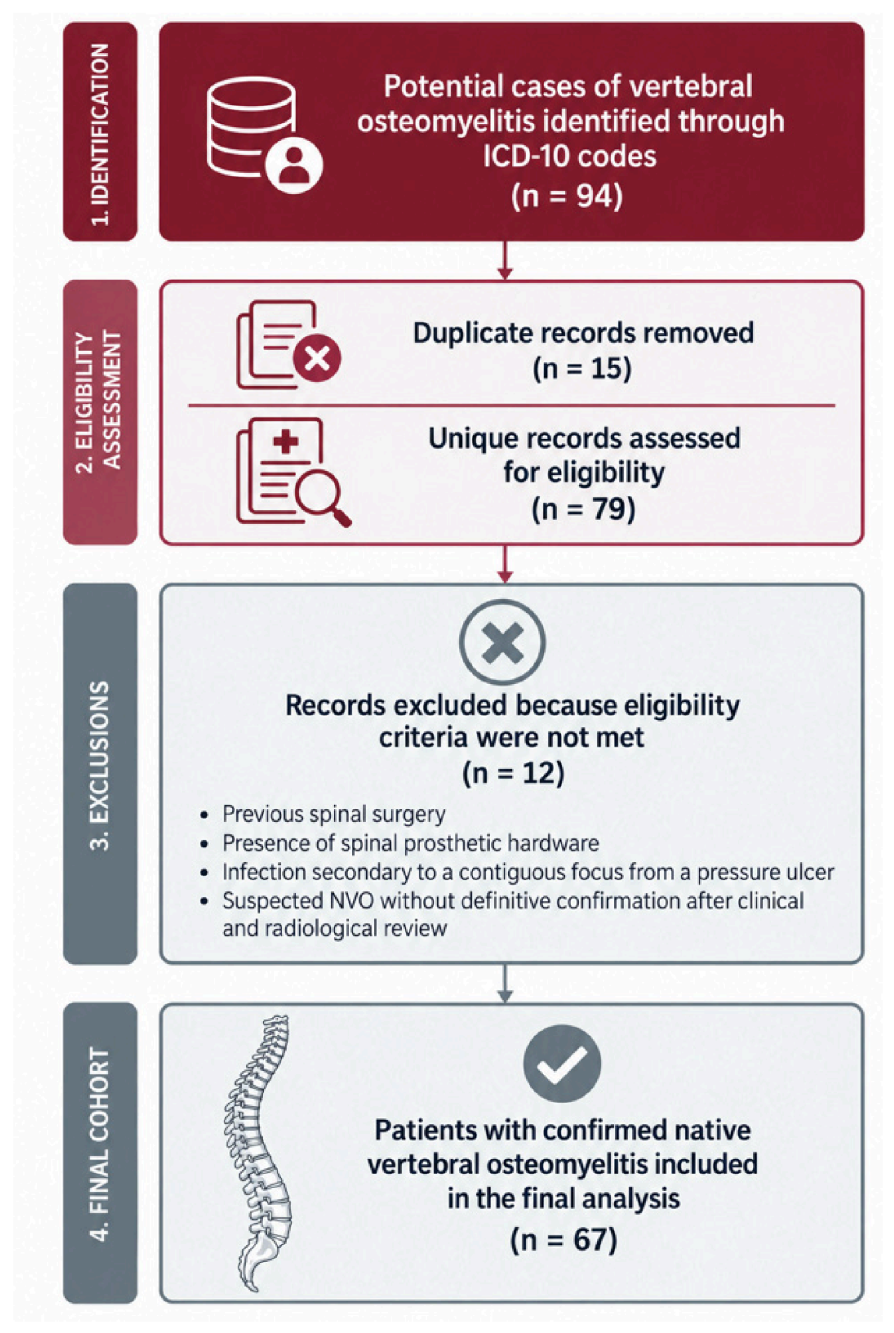
Flow diagram of patient selection and inclusion in the study cohort.

Patient selection and data validation followed a structured multistep process: First, the initial identification of cases using the ICD-10 codes by the Clinical Documentation Department. Second, the identification and removal of duplicate records. Third, the screening of cases according to the predefined inclusion and exclusion criteria. Finally, a manual review of the clinical diagnoses recorded in the medical records, variables were extracted from the clinical records, and additional variables were derived by recoding variables originally recorded in free-text format. Data-cleaning procedures included verification of date consistency and assessment of missing or implausible values in key variables (Figure 2). Missing data were minimal, with 99.26% of values available.

### 2.2. Selection Criteria

Patients were eligible for inclusion if they met the following criteria: i. adult patients (Age ≥ 18 years); ii. a confirmed diagnosis of NVO established either by positive microbiological cultures or through a combination of suggestive typical clinical symptoms, elevated acute-phase reactants, and characteristic radiological findings on MRI or CT; iii. admitted to our center during the study period.

Exclusion criteria included: i. Patients with previous spinal surgery. ii. Presence of spinal prosthetic hardware. iii. infection secondary to a contiguous focus originating from a pressure ulcer. iv. Suspected cases without definitive confirmation of NVO after clinical and radiological review or because of insufficient information.

### 2.3. Data Sources and Variable Measurement

Once the raw dataset was received, the research team refined and analyzed the following variables, categorized into the following groups:

Patient sociodemographic: age, sex, rurality and date of birth were extracted from administrative records. Rurality was determined according to the territorial organization of the Salamanca Health Area, which comprises 36 primary healthcare zones. According to the official classification, there are 22 rural zones, covering 315 municipalities (133,050 inhabitants in 2023; median municipality size, 188 inhabitants), and 3 semi-urban zones (Béjar, Ciudad Rodrigo and Santa Marta de Tormes), covering 46 municipalities (50,166 inhabitants; median, 176 inhabitants) and the 11 urban zones are all within the city of Salamanca (143,954 inhabitants). Population figures were obtained from the Spanish National Statistics Institute (INE). Semi-urban zones were grouped with the rural zones, since their main settlements are towns rather than urban centers, and are surrounded by small rural municipalities, so that 25 zones (361 municipalities, 183,216 inhabitants) were considered rural. Patients assigned to rural zones were classified as rural, and those assigned to urban zones as urban. Nursing home residents were considered a separate category because of their distinct clinical and healthcare context. As an approximation of geographical access, the road distance and driving time from each municipality to the University Hospital of Salamanca, the reference hospital, were estimated using the Open Source Routing Machine (OSRM) public routing service (https://project-osrm.org, accessed on 15 October 2026) using OpenStreetMap road data, from the main settlement of each municipality and road distances and driving times from urban primary healthcare centers to the hospital were obtained using Google Maps (https://maps.google.com/, accessed on 15 October 2026) (Supplementary material Table S2). Municipalities in rural zones were located a median road distance of 59.4 km (range, 5.8–143.0) from the hospital, with a median estimated driving time of 53 min (range, 11–109). Primary healthcare centers in urban zones were located at a median road distance of 2.5 km (range, 1.7–3.3), with a median estimated driving time of 8 min (range, 5–10). These estimates describe the geographical context and do not represent individual patients’ actual journeys.

Baseline clinical variables: Smoking status, presence of comorbidities, and multimorbidity previously assessed and coded by the physicians were considered.

Comorbidities: Comorbidities were obtained from the previously documented diagnoses recorded in the electronic medical records and were selected. The most common comorbidities potentially related to the development of NVO were selected, including pneumonia, chronic kidney disease, infective endocarditis, diabetes mellitus, and urinary tract infection.

Disease-related characteristics: The initial presenting symptom was recorded. Microbiological identification was assessed according to whether a causative microorganism was isolated and, when available, the microorganism identified was recorded. Finally, the affected spinal segment was also documented.

Outcomes: The primary outcome was the presence of complications at admission directly related to NVO as a binary variable (yes/no). Complications were assessed by the physicians without consideration of the patient’s residential origin and subsequently classified into four non-mutually exclusive categories: abscess-related complications, including psoas, paravertebral, prevertebral, epidural and intervertebral abscesses; neurological complications, including paraparesis, paraplegia, myelopathy and radicular deficits; structural complications, including vertebral collapse or fracture, spinal instability, spinal stenosis and spinal cord compression; and systemic complications, including sepsis, septic shock and other systemic manifestations attributable to the infectious process. The composite outcome was used because the study aimed to assess whether rural residence was associated with the presence of any NVO-related complication at admission, rather than with a specific complication type. For the main analysis, each patient was classified as having complications if at least one component was present, regardless of the number or type of complications. Given the limited sample size, this approach focused the analysis on the overarching study question. Component-specific frequencies were reported separately to describe the composition of the outcome. The composite outcome was not intended to imply equivalent clinical severity across different categories, and associations with it should not be interpreted as applying to every individual component.

Secondary outcomes included the need for surgical intervention as a binary variable (yes/no) and length of hospital stay, measured in days from admission to discharge.

Regarding the pathogenetic microbiological diagnosis, a systematic tissue sampling collection was performed in all patients through percutaneous CT-guided biopsy or intraoperatively in those cases requiring surgical intervention. The samples were sent to the hospital microbiology laboratory and processed in accordance with the standard institutional protocols, including aerobic and anaerobic cultures. Microbiological confirmation was the isolation of a pathogenic microorganism in a clinically and radiologically compatible context.

In those cases where the pathogen could not be isolated, the diagnosis was based on compatible clinical and radiological criteria.

All patients included in the study received empiric or targeted antibiotic therapy. Surgical intervention was indicated based on predefined clinical and radiological criteria, including the absence of pathogen identification; progressive neurological deterioration due to spinal cord or root compression; mechanical spinal instability; the presence of abscesses requiring drainage; and the failure of conservative therapy.

### 2.4. Study Sample Size

An initial cohort of 94 potential cases was identified based on the diagnostic codes compatible with Native Vertebral Osteomyelitis.

A non-probabilistic consecutive sampling was used, including all consecutive patients diagnosed with NVO who met the eligibility criteria during the specified study period. From 94 initially identified cases through a retrospective review of medical records, 15 were excluded as duplicate records and 12 records were excluded because they did not meet the eligibility criteria. Finally, a total of 67 patients with confirmed native vertebral osteomyelitis were included in the final analysis.

A sample size calculation was performed a posteriori using the method of Hsieh et al. (1998) [25] for logistic regression with a binary exposure, to assess the adequacy of the available sample size. The calculation assumed an odds ratio of 5 for the association between rural residence and NVO-related complications, a baseline complication probability of 0.463, a rural proportion of 0.388 and an R^2^ of 0.052 between rurality and the other covariates. With a two-sided significance level of 0.05 and 80% power, the calculation indicated a required sample size of 65 subjects. The sample size available (*n* = 67), was therefore sufficient to detect an odds ratio of approximately 5 or greater.

### 2.5. Statistical Analysis

Statistical analyses were performed using IBM SPSS Statistics, version 28, and R Software, version 4.6.0. Descriptive analyses were conducted using absolute frequencies and percentages for qualitative variables and means with standard deviations or medians and interquartile ranges for quantitative variables, depending on data distribution. The normality of quantitative variables was assessed using the Shapiro–Wilk test.

For inferential analyses, categorical variables were compared using contingency tables. For 2 × 2 tables, Fisher’s exact test was used when at least one expected cell count was less than 5. For contingency tables larger than 2 × 2, the likelihood-ratio chi-square test was used when expected counts were less than 5. In all other cases, the Pearson chi-square test was applied.

Continuous variables were compared using a parametric Student’s *t*-test or ANOVA for more than two groups, when normality assumptions were met; otherwise, a nonparametric Mann–Whitney U test or Kruskal–Wallis test for more than two groups was used. The significance level was set at α = 0.05. In all analyses, *p*-values ≤ 0.05 were considered statistically significant.

Finally, a multivariable binary logistic regression model was fitted to assess factors independently associated with the presence of NVO-related complications, the primary outcome. In the main model, age, sex, rurality and multimorbidity were included simultaneously based on their clinical relevance and relationship with the study objective. Age was retained as a continuous variable to preserve information and avoid both a potential loss of statistical power and the additional parameters required by categorization into multiple age groups. Rurality was considered the main exposure of interest, while age, sex and multimorbidity were included as the main potential confounders, as they capture demographic and baseline clinical characteristics that could influence the association between rurality and NVO-related complications. The number of covariates was deliberately restricted to maintain model parsimony given the available sample size. Multimorbidity was selected as the variable to capture the overall burden of morbidity and partially contains information about diabetes mellitus and chronic kidney disease, which are well-known prognostic factors in NVO. Individual prognostic variables that were collected, such as pneumonia, chronic kidney disease, infective endocarditis, diabetes mellitus, urinary tract infection, smoking status, affected spinal segment and microbiological etiology, were deliberately not included independently in the main model to preserve model parsimony and avoid further reducing the events-per-variable ratio and statistical power. With 38 patients experiencing complications and four predictor parameters, the events-per-variable (EPV) ratio was 9.5. Although this was slightly below the conventional recommendation of 10 EPV supported by Peduzzi et al. [26], studies by Vittinghoff and McCulloch found that problems involving bias, confidence interval coverage, and type I error with 5–9 EPV were generally uncommon and, even in the worst instances, were not severe and were usually comparable to those observed with 10–16 EPV [27].

Two sensitivity analyses were performed to examine the consistency and stability of the main model results. First, the multivariable logistic regression model was repeated after excluding nursing home residents to determine whether their inclusion in the non-rural group affected the estimates. Second, Firth’s penalized logistic regression model was fitted to reduce potential small-sample bias. Both models included the same variables as the main model.

Model fit was assessed using the Hosmer–Lemeshow goodness-of-fit test, the omnibus test of model coefficients, and Cox and Snell’s and Nagelkerke’s pseudo-R^2^ coefficients.

A secondary exploratory model was fitted separately to assess the association between the recorded individual comorbidities and the development of NVO-related complications, serving as a supplementary analysis to the main model.

All variables included in each model were entered simultaneously, without automated variable-selection procedures. The strength of the adjusted associations was quantified using odds ratios (ORs) with their corresponding 95% confidence intervals (95% CIs).

## 3. Results

### 3.1. Demographic and Clinical Baseline

A total of 67 patients diagnosed with native vertebral osteomyelitis were included in the study. The mean age was 67.84 years (SD = 18.22), with individual ages ranging from 18 to 94. Males accounted for 70.1% of the sample (*n* = 47), and females 29.9% (*n* = 20). In terms of residential origin, 50.7% of patients were from urban settings (*n* = 34), 38.8% from rural areas (*n* = 26), and 10.4% were nursing home residents (*n* = 7).

Regarding presenting symptoms, pain was the most frequently reported, occurring in 70.1% of patients (*n* = 47), followed by fever in 38.8% (*n* = 26), malaise or fatigue in 9.0% (*n* = 6), dyspnea in 9.0% (*n* = 6), paraparesis in 4.5% (*n* = 3), paresthesia in 1.5% (*n* = 1), vomiting in 1.5% (*n* = 1), and urinary symptoms in 1.5% (*n* = 1). One patient was asymptomatic at presentation. Patients could present with more than one symptom.

The lumbar spine was the most frequently affected anatomical region, occurring in 67.2% of patients (*n* = 45), followed by the thoracic region in 37.3% (*n* = 25), the cervical region in 9.0% (*n* = 6), and the sacral region in 9.0% (*n* = 6). More than one anatomical region could be affected in the same patient.

### 3.2. Etiological and Comorbidity Profile

Overall, 40.3% of patients (*n* = 27) were classified as multimorbid. At least one comorbidity was identified in 88.1% (*n* = 59). Diabetes mellitus was recorded in 25.4% of patients (*n* = 17) and chronic kidney disease in 17.9% (*n* = 12). Infective endocarditis was diagnosed in 19.4% (*n* = 13), urinary tract infection in 17.9% (*n* = 12), and pneumonia in 10.4% (*n* = 7). Active smokers accounted for 20.9% of the cohort (*n* = 14).

Microbiological diagnosis was achieved in 46.3% of patients (*n* = 31). *Staphylococcus aureus* was the most frequently isolated microorganism, identified in 16.4% of cases (*n* = 11), followed by Enterobacteriaceae, including *Klebsiella pneumoniae*, *Citrobacter koseri*, and *Enterobacter spp.*, which collectively were identified in 11.9% (*n* = 8). No pathogen was identified in the remaining 53.7% of patients (*n* = 36).

### 3.3. Management and Outcomes

The mean hospital stay was 31.69 days (SD = 20.86), with durations ranging from 1 to 87 days. All patients in the cohort received antibiotic treatment as part of their initial management. Surgical intervention was required in 47.8% of cases (*n* = 32), while the remaining 52.2% (*n* = 35) were managed conservatively. Readmission occurred in 19.4% of patients (*n* = 13); among those readmitted, 69.2% (*n* = 9) underwent a subsequent surgical procedure.

NVO-related complications were documented in 56.7% of cases (*n* = 38). The most common were abscess-related complications, observed in 40.3% (*n* = 27), followed by neurological complications in 16.4% (*n* = 11), systemic complications in 9.0% (*n* = 6), and structural complications in 6.0% (*n* = 4). Individual patients could experience complications from more than one category.

### 3.4. Comparative Analysis by Rurality

Comparative analyses according to rurality revealed the following main findings (Table 1 and Table 2). NVO-related complications were significantly more frequent among rural than non-rural patients (73.1% vs. 46.3%; *p* = 0.031). This difference remained significant when rural patients were compared specifically with urban patients (73.1% vs. 44.1%; *p* = 0.025). When urban patients, rural patients and nursing home residents were compared as three separate groups, the difference did not reach statistical significance (44.1%, 73.1%, and 57.1%, respectively; *p* = 0.087).

Multimorbidity was less frequent among rural patients than non-rural patients (26.9% vs. 48.8%), although this difference was not statistically significant (*p* = 0.075). No other statistically significant differences were observed in the pairwise comparisons between rural and non-rural or rural and urban patients.

### 3.5. Multivariable Logistic Regression Models of Factors Associated with NVO-Related Complications

**Figure 3:**
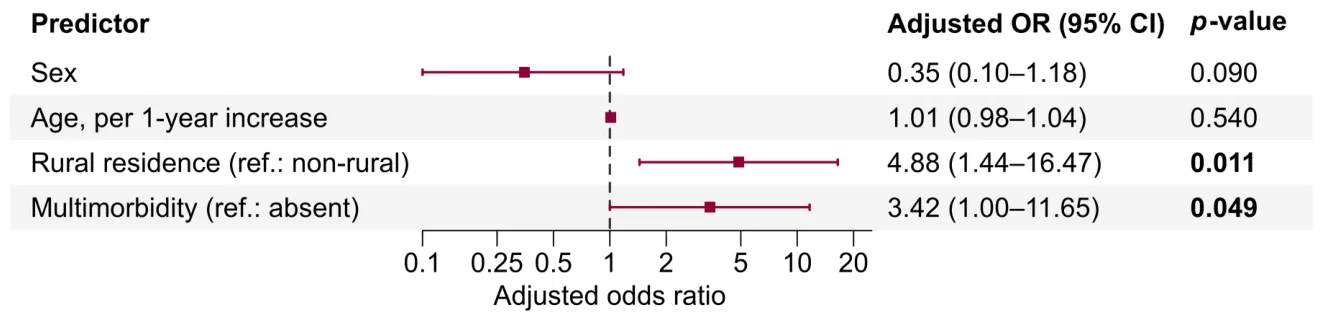
Factors associated with NVO-related complications.

A first multivariable binary logistic regression model including rurality, sex, age, and multimorbidity was fitted to examine their associations with NVO-related complications. Rurality and multimorbidity were independently associated with NVO-related complications. Rural patients had 4.88 times the odds of complications compared with non-rural patients (aOR = 4.88; 95% CI, 1.44–16.47; *p* = 0.011). Multimorbid patients had 3.42 times the odds of complications compared with non-multimorbid patients (aOR = 3.42; 95% CI, 1.00–11.65; *p* = 0.049). No significant associations were observed for sex (aOR = 0.35; 95% CI, 0.10–1.18; *p* = 0.090) or age (aOR = 1.01; 95% CI, 0.98–1.04; *p* = 0.540) (Figure 3).

Regarding the goodness of fit of the model, the Hosmer–Lemeshow test was not statistically significant (χ^2^ = 9.601; df = 8; *p* = 0.294), indicating no evidence of lack of fit. The pseudo-R^2^ coefficients were 0.255 for Nagelkerke’s R^2^ and 0.190 for Cox and Snell’s R^2^. The overall model was statistically significant (omnibus test: χ^2^ = 14.098; df = 4; *p* = 0.007).

Finally, two sensitivity analyses were performed to assess the robustness of the main model results. First, the multivariable logistic regression model was repeated after excluding nursing home residents to determine whether the model estimates were influenced by their inclusion in the non-rural group. Second, Firth’s penalized logistic regression was fitted to address potential small-sample bias.

After excluding nursing home residents, rurality (aOR = 5.83; 95% CI, 1.60–21.27; *p* = 0.008) and multimorbidity (aOR = 5.00; 95% CI, 1.28–19.53; *p* = 0.021) were independently associated with NVO-related complications. No significant associations were observed for sex (aOR = 0.63; 95% CI, 0.17–2.36; *p* = 0.491) or age (aOR = 1.01; 95% CI, 0.98–1.05; *p* = 0.418).

In the Firth penalized model, rurality (aOR = 4.21; 95% CI, 1.40–14.32; *p* = 0.010) remained independently associated with NVO-related complications. No significant associations were observed for sex (aOR = 0.38; 95% CI, 0.12–1.19; *p* = 0.096), age (aOR = 1.01; 95% CI, 0.98–1.04; *p* = 0.555) or multimorbidity (aOR = 3.08; 95% CI, 0.99–10.49; *p* = 0.052).

**Figure 4:**
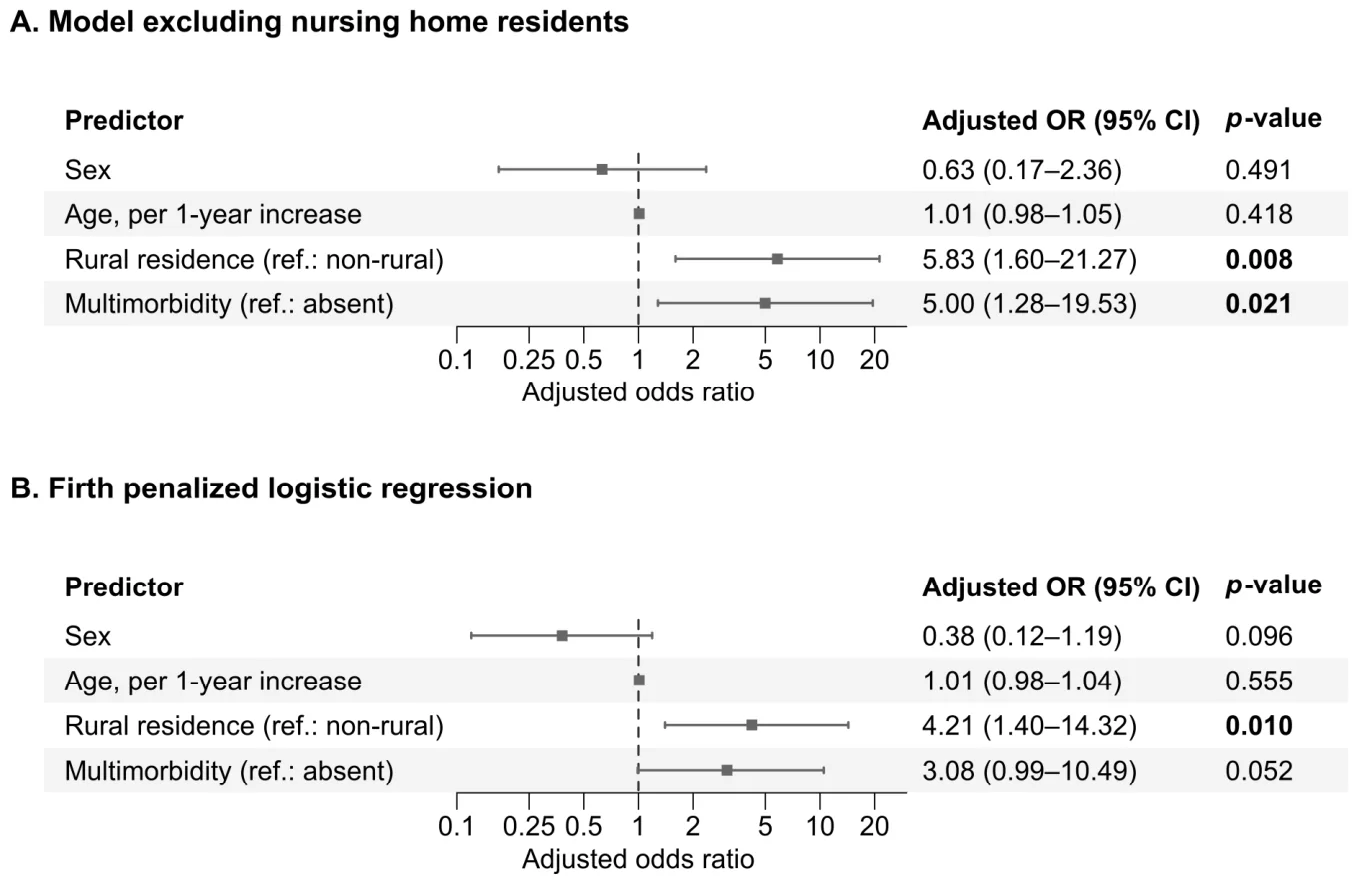
Factors associated with NVO-related complications. Sensitivity analyses: (**A**) Model excluding nursing home residents. (**B**) Firth penalized logistic regression model.

The association between rurality and NVO-related complications remained consistent across both sensitivity analyses, whereas multimorbidity was significantly associated with NVO-related complications in the main model and after nursing home residents were excluded (Figure 4).

A second exploratory binary logistic regression model was fitted to examine the associations between individual comorbidities and NVO-related complications.

This model included pneumonia, chronic kidney disease, infectious endocarditis, diabetes mellitus and urinary tract infection. No statistically significant associations were observed between the individual comorbidities and NVO-related complications (*p* > 0.05).

## 4. Discussion

### 4.1. Key Results and Interpretation

The main finding of this study is that rurality was independently associated, after adjustment for age, sex and multimorbidity, with almost five times the odds of having NVO-related complications compared with non-rural patients. Multimorbidity was also independently associated with more than three times the odds of developing NVO-related complications compared with patients without multimorbidity. No statistically significant associations were observed for sex or age. The association of rurality remained consistent after excluding nursing home residents from the non-rural group and after applying Firth’s penalized logistic regression. The association with multimorbidity did not reach statistical significance in the Firth-penalized model. These sensitivity analyses suggest that the observed association with rurality was not solely explained by the inclusion of nursing home residents or by the estimation method used. However, given the wide confidence intervals, its magnitude remains uncertain.

In this context, rural patients showed a significantly higher frequency of NVO-related complications than both urban and non-rural patients. However, when urban patients, rural patients and nursing home residents were compared as three separate groups, the difference did not reach statistical significance, which should be interpreted cautiously given the small size of the nursing home subgroup.

Multimorbidity was descriptively less frequent among rural than non-rural patients; hence, the higher frequency of complications observed among rural patients was not accompanied by a higher prevalence of recorded multimorbidity. Thus, rurality may act as an indicator of underlying factors, such as socioeconomic context, limited access to timely and specialized care, including diagnostic imaging, a smaller number of healthcare professionals, and poorer transportation and longer travel distances [11,28,29]. Each of these factors may contribute to delays in the recognition of NVO and its complications. Specifically, socioeconomic context may influence how promptly patients seek care [29], limited access to specialized care and imaging may delay confirmation of the diagnosis [12,13], a smaller number of healthcare professionals and capacity constraints in primary care may reduce the likelihood of early suspicion [10,11], and poorer transportation and longer travel distances may delay both the initial consultation and referral to a tertiary center [10,30]. The contribution of these factors to the observed association could not be established in this study and further investigation is required to explore it. Clinically, these findings suggest that rural patients may present with a greater burden of NVO-related complications at admission that is not accounted for by age, sex or recorded multimorbidity. Rurality may reflect contextual factors relevant to clinical presentation, which could not be identified in the present study, and these results should therefore be considered hypothesis-generating. In the meantime, it may be reasonable to maintain a lower threshold of clinical suspicion for NVO in primary care settings from rural areas when patients present with symptoms and clinical features suggestive of NVO, and to consider prompt MRI assessment in accordance with clinical guidelines [6]. Rapid referral pathways from rural areas to the reference hospital could also be explored to determine whether they shorten diagnostic intervals and reduce complications.

To the best of our knowledge, no previous studies have investigated in depth the relationship between rurality and NVO or, more specifically, the association between rurality and NVO-related complications. Consequently, direct comparison with our findings is limited. Nevertheless, the sparse and heterogeneous evidence available allows for some general comparisons. Ribeira et al. (2008) [20] and Guo et al. (2025) [19] reported higher proportions of rural patients in spinal infection cohorts than the proportion observed in our study, reaching 71% and 63%, respectively, compared with 38.8% in our cohort. Similarly, Yilboudo et al. (2002) [24] reported that 90% of patients with NVO-related neurological manifestations requiring surgical intervention came from rural areas, in line with the greater burden of complications observed among rural patients in our cohort. However, differences in disease etiology, clinical context and patient selection limit the comparability of these descriptive observations with the present findings.

Regarding multimorbidity, our findings are aligned with previous evidence linking a greater comorbidity burden to poorer outcomes in patients with NVO. Nolla et al. (2002) [15] noted that over 40% of patients in their cohort exhibited complex comorbidity profiles, while Loibl et al. (2014) [31] reported multimorbidity rates of up to 82.6% in their cohort, which were also associated with increased mortality. This may suggest that the cumulative burden of multiple conditions is more relevant than any individual comorbidity considered separately. These findings are compatible with multimorbidity being a marker of clinical vulnerability, although they do not establish that cumulative comorbidity is more informative than individual conditions.

Regarding the profile of our cohort, demographically, the mean age was approximately 68 years, reflecting established epidemiological trends. Ziarko et al. (2023) [32] and Hsieh et al. (2004) [33] emphasized how the comorbidity burden in older adults demonstrates heightened susceptibility to vertebral infections. A male predominance was observed, consistent with Urrutia and Fuentealba (2020) [8], who documented male-to-female ratios ranging from 2:1 to 5:1.

Clinically, pain was the most common initial symptom, consistent with the systematic review by Mylona et al. (2009) [9], where spinal pain was reported in up to 86% of patients. Fever was less frequent in our cohort and has been variably reported in previous studies. For instance, Carragee (1997) [34] found fever in only 16.2% of patients, whereas Pigrau et al. (2005) [35] observed it in up to 80% of their specific cohort. This marked clinical variability, later synthesized in the systematic review by Mylona et al. (2009) [9], which reported an average prevalence of 60%, indicates that the absence of fever does not rule out the disease.

The lumbar spine was the most frequently affected region, observed in 67.2% of cases, in agreement with the findings of Grammatico et al. (2008) [36] and Gentile et al. (2019) [16], who attributed this lumbar predominance to its greater mobility and extensive vascular supply, which facilitates hematogenous seeding. Neurological complications, including spinal cord compression and paraparesis, occurred in a minority of patients, as previously noted by Hsieh et al. (2004) [33], whose study described complication rates between 6% and 17%.

Microbiological confirmation was achieved in 46.3% of patients, similar to the 43.8% reported by Yu et al. (2020) [37] but higher than the rates below 25% described by Hsieh et al. (2004) [33]. The most commonly identified microorganism was *Staphylococcus aureus*, followed by Enterobacteriaceae species, consistent with Gao et al. (2024) [14], who identified *S. aureus* as the predominant pathogen, particularly in cases of hematogenous spread.

Infective endocarditis (IE) was diagnosed in 19.4% of our patients, a proportion exceeding the 2–10% reported by Behmanesh et al. (2019) [38] and the 12% observed in the study by Morimoto et al. (2022) [39].

All patients in the present study received antibiotic therapy. Current clinical guidelines, specifically the IDSA recommendations published by Berbari et al. (2015) [6], recommend a total antibiotic treatment duration of approximately six weeks, administered either parenterally or orally with high bioavailability, and adjusted according to the patient’s clinical and microbiological response. However, early administration of empirical antimicrobials is well-known to be associated with culture-negative NVO. Surgical intervention was required in 47.8% of cases, primarily for progressive neurological deterioration or mechanical instability. These indications align with the surgical strategies described by Lee et al. (2022) [40], in which intervention is reserved for patients failing conservative therapy or presenting with spinal cord compression.

NVO-related complications at admission were frequent, observed in 56.7% of patients. Abscess-related complications were the most prevalent (40.3%), followed by neurological complications (16.4%). This distribution parallels findings by Gentile et al. (2019) [16], who synthesized various cohorts to note abscess formation in 30.4% of NVO cases and neurological complications in 27.8% of NVO cases.

### 4.2. Limitations

This study has several limitations that should be considered. First, the single-center design may limit the generalizability of the findings. Second, the retrospective design of the study may introduce information bias. Third, the sample size was relatively limited, which may have resulted in wide confidence intervals and imprecise estimates. This concern was particularly relevant to the secondary exploratory model, which included more predictors and had a lower EPV ratio. Non-significant findings in this model should not be interpreted as evidence of no association. Limited statistical power may have contributed to these results. In addition, the small number of nursing home residents makes it appropriate to interpret the results involving this group cautiously. Finally, some potential confounding factors may not have been accounted for when fitting the main model. The number of variables included was limited to preserve model parsimony and avoid overfitting. On the one hand, some variables collected in our study that may influence clinical outcomes, such as the causative pathogen, could not be included because no pathogen was identified in a considerable number of cases. Other individual variables, such as pneumonia, chronic kidney disease, infective endocarditis, diabetes mellitus, urinary tract infection, smoking status, affected spinal segment and microbiological etiology, were deliberately not included in the main model to preserve model parsimony and avoid further reducing the events-per-variable ratio and statistical power. On the other hand, other unmeasured variables, such as symptom-to-consultation, symptom-to-diagnosis and referral intervals, socioeconomic context, limited access to timely and specialized care, a smaller number of healthcare professionals, and poorer transportation and longer travel distances, may partly explain the association between rurality and NVO-related complications. Therefore, residual confounding cannot be excluded, and further investigation is required.

### 4.3. Generalisability

Regarding external validity, our study population reflects a real-world cohort of adult patients with NVO treated at a tertiary university hospital. However, the single-center design may limit the generalizability of our findings to other settings or healthcare systems. Additionally, variations in geographical characteristics, healthcare accessibility, referral pathways, diagnostic and therapeutic management and institutional practices should be considered when interpreting these results.

## 5. Conclusions

In conclusion, these findings suggest that rurality may represent a relevant factor in the clinical course of NVO. Further multicenter studies are required to confirm this association and explore its underlying mechanisms.

## Figures and Tables

**Table 1:** Demographic characteristics, etiology, and clinical outcomes in patients with NVO: comparisons across urban, rural, and nursing home groups and between urban and rural patients.

Domain/Variable	Rural (*n* = 26)	Urban (*n* = 34)	Nursing Homes (*n* = 7)	*p* (Urban vs. Rural)	*p* (Three-Group Comparison)
**Biodemographic Parameters**					
Age (mean ± SD)	68.27 ± 15.51	66.35 ± 20.49	73.43 ± 16.94	0.893	0.595
Active smoking	19.2% (*n* = 5)	23.5% (*n* = 8)	14.3% (*n* = 1)	0.689	0.871
Initial presentation					
*Pain*	64.0% (*n* = 16)	84.8% (*n* = 28)	50.0% (*n* = 3)	0.066	0.067
*Fever*	36.0% (*n* = 9)	45.5% (*n* = 15)	33.3% (*n* = 2)	0.469	0.607
*Malaise and fatigue*	8.0% (*n* = 2)	9.1% (*n* = 3)	16.7% (*n* = 1)	1.000	0.669
*Paresthesia*	4.0% (*n* = 1)	0.0% (*n* = 0)	0.0% (*n* = 0)	0.431	0.484
*Paraparesis*	12.0% (*n* = 3)	0.0% (*n* = 0)	0.0% (*n* = 0)	0.075	0.120
*Vomiting*	4.0% (*n* = 1)	0.0% (*n* = 0)	0.0% (*n* = 0)	0.431	0.484
*Dyspnea*	4.0% (*n* = 1)	3.0% (*n* = 1)	66.7% (*n* = 4)	1.000	<0.001 *
*Urinary symptoms*	0.0% (*n* = 0)	0.0% (*n* = 0)	16.7% (*n* = 1)	1.000	0.094
*Asymptomatic*	0.0% (*n* = 0)	3.0% (*n* = 1)	0.0% (*n* = 0)	1.000	1.000
Segment affected					
*Cervical*	11.5% (*n* = 3)	5.9% (*n* = 2)	14.3% (*n* = 1)	0.644	0.557
*Thoracic*	34.6% (*n* = 9)	35.3% (*n* = 12)	57.1% (*n* = 4)	0.956	0.552
*Lumbar*	65.4% (*n* = 17)	67.6% (*n* = 23)	71.4% (*n* = 5)	0.854	1.000
*Sacral*	0.0% (*n* = 0)	14.7% (*n* = 5)	14.3% (*n* = 1)	0.063	0.084
**Etiological and comorbidity profile**					
Comorbidity present	92.3% (*n* = 24)	82.4% (*n* = 28)	100.0% (*n* = 7)	0.446	0.370
Multimorbidity	26.9% (*n* = 7)	47.1% (*n* = 16)	57.1% (*n* = 4)	0.112	0.175
Diabetes mellitus	26.9% (*n* = 7)	17.6% (*n* = 6)	57.1% (*n* = 4)	0.387	0.103
Chronic kidney disease	19.2% (*n* = 5)	14.7% (*n* = 5)	28.6% (*n* = 2)	0.733	0.658
Pneumonia	11.5% (*n* = 3)	8.8% (*n* = 3)	14.3% (*n* = 1)	1.000	0.861
Urinary tract infection	11.5% (*n* = 3)	17.6% (*n* = 6)	42.9% (*n* = 3)	0.719	0.162
Infective endocarditis	19.2% (*n* = 5)	20.6% (*n* = 7)	14.3% (*n* = 1)	1.000	1.000
Pathogen identified	50.0% (*n* = 13)	41.2% (*n* = 14)	57.1% (*n* = 4)	0.496	0.725
**Management and outcomes**					
Hospital stay (days; mean ± SD)	30.31 ± 16.83	31.09 ± 21.00	39.71 ± 33.08	0.923	0.930
Surgical intervention	53.8% (*n* = 14)	44.1% (*n* = 15)	42.9% (*n* = 3)	0.603	0.728
Readmission	19.2% (*n* = 5)	14.7% (*n* = 5)	42.9% (*n* = 3)	0.733	0.233
Second intervention	11.5% (*n* = 3)	14.7% (*n* = 5)	14.3% (*n* = 1)	1.000	1.000
NVO-related complications	73.1% (*n* = 19)	44.1% (*n* = 15)	57.1% (*n* = 4)	0.025 *	0.087
Type of complication					
*Abscess-related*	50.0% (*n* = 13)	32.4% (*n* = 11)	42.9% (*n* = 3)	0.167	0.421
*Neurological*	26.9% (*n* = 7)	11.8% (*n* = 4)	0.0% (*n* = 0)	0.182	0.203
*Structural*	7.7% (*n* = 2)	2.9% (*n* = 1)	14.3% (*n* = 1)	0.574	0.311
*Systemic*	3.8% (*n* = 1)	8.8% (*n* = 3)	28.6% (*n* = 2)	0.626	0.139

* Data are presented as % (*n*) for categorical variables and mean ± standard deviation (SD) for continuous variables. Non-rural includes urban patients and nursing home residents. Pairwise comparisons (rural vs. urban) were performed using the independent-samples *t*-test or Mann–Whitney U test for continuous variables and Pearson’s chi-square test or Fisher’s exact test for categorical variables, as appropriate. Three-group comparisons among urban, rural, and nursing home patients were performed using one-way ANOVA or the Kruskal–Wallis test for continuous variables and Pearson’s chi-square test or the likelihood-ratio chi-square test for categorical variables, as appropriate. Statistically significant results (*p* < 0.05) are shown in bold and marked with an asterisk (*). Bold headings in the first column distinguish variable domains.

**Table 2:** Demographic characteristics, etiology, and clinical outcomes in patients with NVO: comparison between non-rural and rural patients.

Domain/Variable	Non-Rural (*n* = 41)	Rural (*n* = 26)	*p* (Non-Rural vs. Rural)
**Biodemographic Parameters**			
Age (mean ± SD)	67.56 ± 19.92	68.27 ± 15.51	0.847
Active smoking	22.0% (*n* = 9)	19.2% (*n* = 5)	0.790
Initial presentation			
*Pain*	79.5% (*n* = 31)	64.0% (*n* = 16)	0.246
*Fever*	43.6% (*n* = 17)	36.0% (*n* = 9)	0.546
*Malaise and fatigue*	10.3% (*n* = 4)	8.0% (*n* = 2)	1.000
*Paresthesia*	0.0% (*n* = 0)	4.0% (*n* = 1)	0.391
*Paraparesis*	0.0% (*n* = 0)	12.0% (*n* = 3)	0.055
*Vomiting*	0.0% (*n* = 0)	4.0% (*n* = 1)	0.391
*Dyspnea*	12.8% (*n* = 5)	4.0% (*n* = 1)	0.391
*Urinary symptoms*	2.6% (*n* = 1)	0.0% (*n* = 0)	1.000
*Asymptomatic*	2.6% (*n* = 1)	0.0% (*n* = 0)	1.000
Segment affected			
*Cervical*	7.3% (*n* = 3)	11.5% (*n* = 3)	0.670
*Thoracic*	39.0% (*n* = 16)	34.6% (*n* = 9)	0.716
*Lumbar*	68.3% (*n* = 28)	65.4% (*n* = 17)	1.000
*Sacral*	14.6% (*n* = 6)	0.0% (*n* = 0)	0.074
**Etiological and comorbidity profile**			
Comorbidity present	85.4% (*n* = 35)	92.3% (*n* = 24)	0.469
Multimorbidity	48.8% (*n* = 20)	26.9% (*n* = 7)	0.075
Diabetes mellitus	24.4% (*n* = 10)	26.9% (*n* = 7)	1.000
Chronic kidney disease	17.1% (*n* = 7)	19.2% (*n* = 5)	1.000
Pneumonia	9.8% (*n* = 4)	11.5% (*n* = 3)	1.000
Urinary tract infection	22.0% (*n* = 9)	11.5% (*n* = 3)	0.343
Infective endocarditis	19.5% (*n* = 8)	19.2% (*n* = 5)	0.977
Pathogen identified	43.9% (*n* = 18)	50.0% (*n* = 13)	0.626
**Management and outcomes**			
Hospital stay (days; mean ± SD)	32.56 ± 23.21	30.31 ± 16.83	0.944
Surgical intervention	43.9% (*n* = 18)	53.8% (*n* = 14)	0.427
Readmission	19.5% (*n* = 8)	19.2% (*n* = 5)	0.977
Second intervention	14.6% (*n* = 6)	11.5% (*n* = 3)	1.000
NVO-related complications	46.3% (*n* = 19)	73.1% (*n* = 19)	0.031 *
Type of complication			
*Abscess-related*	34.1% (*n* = 14)	50.0% (*n* = 13)	0.197
*Neurological*	9.8% (*n* = 4)	26.9% (*n* = 7)	0.092
*Structural*	4.9% (*n* = 2)	7.7% (*n* = 2)	0.638
*Systemic*	12.2% (*n* = 5)	3.8% (*n* = 1)	0.392

* Data are presented as % (*n*) for categorical variables and mean ± standard deviation (SD) for continuous variables. Non-rural includes urban patients and nursing home residents. Pairwise comparisons (non-rural vs. rural) were performed using the independent-samples *t*-test or Mann–Whitney U test for continuous variables and Pearson’s chi-square test or Fisher’s exact test for categorical variables, as appropriate. Statistically significant results (*p* < 0.05) are shown in bold and marked with an asterisk (*). Bold headings in the first column distinguish variable domains.

## Data Availability

The data presented in this study are available from the corresponding author upon reasonable request and subject to institutional review. The data are not publicly available due to privacy and ethical restrictions associated with patient-level clinical data.

## Supplementary Materials

The following supporting information can be downloaded at https://www.mdpi.com/article/10.3390/jcm15218455/s1: Table S1: STROBE checklist; Table S2: Population, road distance and driving time from each municipality to the University Hospital of Salamanca (reference hospital), by primary healthcare zone of the Salamanca Health Area.
