# Supplementary material for: Clinical Complexity in Native Vertebral Osteomyelitis: Exploring the Role of Rurality—A Retrospective Cohort Study
Source: J Clin Med. 2026 Oct 30;15(21):8455. doi: 10.3390/jcm15218455 (PMC13763961; doi:10.3390/jcm15218455)
Supplement: Supplementary file 1 [file jcm-15-08455-s001.zip › Supplementary_Table_S2-edit.pdf]

Supplementary Table S2. Population, road distance and driving time from each municipality to the University Hospital of Salamanca (reference hospital) by primary healthcare zone of the Salamanca Health Area.

Municipality	Population 2023	Distance (km)	Driving time (h:min)
**Rural zones (n = 25 zones; 362 municipalities)**			
**Periurbana Norte (n = 14); median 11.8 km (range 5.8–23.3)**			
Aldealengua	668	11.4	0:22
Aldearrubia	514	17.3	0:31
Almenara de Tormes	299	19.0	0:24
Cabrerizos	4,224	6.6	0:16
Castellanos de Moriscos	2,989	12.2	0:16
Castellanos de Villiquera	683	11.4	0:17
Juzbado	189	23.3	0:29
Monterrubio de Armuña	1,315	9.0	0:16
Moriscos	552	12.5	0:16
San Cristóbal de la Cuesta	1,114	10.1	0:17
San Morales	354	15.9	0:30
Valverdón	267	14.2	0:20
Villamayor	7,539	5.8	0:11
Villares de la Reina	6,576	6.7	0:13
**Santa Marta de Tormes (n = 8); median 15.2 km (range 6.2–26.5)**			
Calvarrasa de Abajo	1,191	13.5	0:18
Calvarrasa de Arriba	597	11.7	0:17
Encinas de Abajo	618	22.7	0:23
Huerta	278	26.5	0:29
Machacón	444	16.9	0:23
Pelabravo	1,323	12.6	0:18
Santa Marta de Tormes	14,683	6.2	0:14
Villagonzalo de Tormes	226	22.6	0:27
**Periurbana Sur (n = 20); median 19.1 km (range 6.6–27.2)**			
Aldeatejada	2,518	10.2	0:19
Arapiles	725	10.9	0:16
Barbadillo	395	22.9	0:24
Carbajosa de la Sagrada	7,555	6.6	0:13
Carrascal de Barregas	1,415	12.4	0:19
Doñinos de Salamanca	2,288	8.8	0:15
El Pino de Tormes	155	16.4	0:27
Florida de Liébana	251	13.2	0:23
Galindo y Perahuy	665	24.8	0:26
Golpejas	148	23.1	0:27
Miranda de Azán	455	11.4	0:18
Monterrubio de la Sierra	152	26.8	0:37
Morille	227	21.0	0:34
Mozárbez	514	17.3	0:35
Parada de Arriba	259	13.9	0:20
Rollán	341	27.2	0:28
San Pedro de Rozados	277	22.2	0:37
San Pedro del Valle	149	22.6	0:29
Vega de Tirados	159	23.3	0:30
Zarapicos	60	21.9	0:27

Municipality	Population 2023	Distance (km)	Driving time (h:min)
**Pedrosillo el Ralo (n = 15); median 21.2 km (range 18.3–32.5)**			
Aldeanueva de Figueroa	254	32.3	0:31
Arcediano	100	18.5	0:26
Cabezabellosa de la Calzada	75	23.6	0:28
Espino de la Orbada	240	31.2	0:33
Gomecello	409	19.6	0:22
La Orbada	181	26.0	0:25
La Vellés	517	20.6	0:23
Negrilla de Palencia	80	18.7	0:26
Pajares de la Laguna	109	24.5	0:24
Palencia de Negrilla	145	18.3	0:26
Parada de Rubiales	249	32.5	0:31
Pedrosillo el Ralo	129	18.6	0:20
Pitiegua	179	26.5	0:32
Tardáguila	192	21.2	0:29
Villaverde de Guareña	140	20.3	0:23
**Calzada de Valdunciel (n = 13); median 27.9 km (range 15.1–37.0)**			
Aldearrodrigo	146	23.4	0:29
Añover de Tormes	89	34.0	0:38
Calzada de Valdunciel	702	15.1	0:20
El Arco	93	25.0	0:30
Forfoleda	208	19.4	0:24
Palacios del Arzobispo	151	34.5	0:40
San Pelayo de Guareña	92	27.9	0:33
Santiz	246	37.0	0:42
Topas	512	30.9	0:32
Torresmenudas	185	20.7	0:26
Valdelosa	417	34.2	0:31
Valdunciel	101	21.1	0:21
Zamayón	191	27.9	0:33
**Villoria (n = 8); median 33.0 km (range 28.5–56.8)**			
Arabayona de Mógica	333	37.4	0:42
Babilafuente	927	28.5	0:30
Cantalpino	817	56.8	0:49
Cordovilla	111	28.6	0:29
El Pedroso de la Armuña	209	35.1	0:40
Moríñigo	87	29.3	0:31
Villoria	1,267	33.1	0:34
Villoruela	738	32.9	0:36
**Alba de Tormes (n = 29); median 34.5 km (range 20.6–56.5)**			
Alba de Tormes	5,058	23.9	0:29
Aldeaseca de Alba	66	29.8	0:37
Anaya de Alba	187	34.5	0:39
Armenteros	256	56.5	1:01
Beleña	277	27.3	0:26
Buenavista	373	26.4	0:26
Chagarcía Medianero	80	49.8	0:54
Coca de Alba	102	36.2	0:37
Éjeme	144	30.4	0:38
Encinas de Arriba	235	29.9	0:34
Fresno Alhándiga	191	33.8	0:30
Gajates	146	40.2	0:52

Municipality	Population 2023	Distance (km)	Driving time (h:min)
Galinduste	394	45.1	0:45
Galisancho	333	41.3	0:41
Garcihernández	418	31.6	0:37
Horcajo Medianero	212	47.5	0:51
La Maya	159	36.3	0:32
Larrodrigo	183	38.2	0:44
Martinamor	79	25.7	0:26
Navales	305	28.8	0:35
Pedraza de Alba	222	39.0	0:50
Pedrosillo de Alba	124	34.5	0:44
Pedrosillo de los Aires	321	42.2	0:42
Pelayos	74	42.1	0:40
Peñarandilla	184	36.6	0:41
Sieteiglesias de Tormes	175	38.4	0:35
Terradillos	3,104	20.6	0:27
Valdecarros	314	34.2	0:43
Valdemierque	58	28.3	0:29
**Matilla de los Caños (n = 12); median 35.6 km (range 22.2–48.8)**			
Aldehuela de la Bóveda	271	36.9	0:30
Calzada de Don Diego	137	22.7	0:21
Canillas de Abajo	75	26.1	0:26
Carrascal del Obispo	192	45.4	0:44
Las Veguillas	274	37.9	0:46
Matilla de los Caños del Río	628	36.5	0:31
Robliza de Cojos	190	30.9	0:26
San Pedro de Rozados	277	22.2	0:37
Sanchón de la Sagrada	36	48.8	0:47
Tabera de Abajo	102	34.7	0:33
Vecinos	226	29.2	0:33
Villalba de los Llanos	113	41.1	0:38
**Ledesma (n = 14); median 46.8 km (range 29.0–64.0)**			
Doñinos de Ledesma	67	36.0	0:40
El Manzano	60	64.0	1:05
Encina de San Silvestre	111	44.3	0:49
Gejuelo del Barro	31	46.6	0:48
La Mata de Ledesma	104	30.3	0:35
Ledesma	1,539	35.1	0:40
Monleras	241	57.5	1:00
Sando	118	47.4	0:48
Santa María de Sando	98	49.5	0:52
Sardón de los Frailes	98	62.9	1:05
Villarmayor	165	29.0	0:33
Villasdardo	23	46.9	0:52
Villaseco de los Gamitos	130	41.4	0:44
Villaseco de los Reyes	296	52.9	0:55
**Linares de Riofrío (n = 13); median 51.9 km (range 41.8–64.2)**			
Barbalos	71	45.0	0:52
El Tornadizo	87	59.8	1:03
Endrinal	203	64.2	0:58
Escurial de la Sierra	233	50.9	0:55
Frades de la Sierra	181	46.1	0:59
Herguijuela del Campo	66	48.8	0:53

Municipality	Population 2023	Distance (km)	Driving time (h:min)
La Sierpe	41	50.8	0:56
Linares de Riofrío	923	51.9	0:53
Membribe de la Sierra	102	41.8	0:52
Monleón	89	60.2	1:01
San Esteban de la Sierra	371	64.2	1:07
San Miguel de Valero	316	57.1	0:59
Valero	301	63.6	1:09
**Peñaranda de Bracamonte (n = 18); median 52.2 km (range 32.3–67.9)**			
Alaraz	437	67.9	1:02
Alconada	140	32.4	0:31
Aldeaseca de la Frontera	254	50.8	0:44
Bóveda del Río Almar	199	53.7	0:48
Cantaracillo	188	50.2	0:41
El Campo de Peñaranda	263	43.5	0:42
Macotera	1,003	58.6	0:51
Malpartida	90	64.5	1:02
Mancera de Abajo	203	54.8	0:50
Nava de Sotrobal	165	44.0	0:46
Paradinas de San Juan	394	53.8	0:44
Peñaranda de Bracamonte	6,079	47.3	0:39
Rágama	204	56.2	0:46
Salmoral	114	59.5	0:54
Santiago de la Puebla	290	61.7	0:55
Tordillos	324	40.9	0:44
Ventosa del Río Almar	104	32.3	0:29
Villar de Gallimazo	195	39.0	0:35
**Cantalapiedra (n = 6); median 55.0 km (range 44.5–60.1)**			
Cantalapiedra	909	60.1	0:52
Palaciosrubios	305	54.2	0:58
Poveda de las Cintas	197	44.5	0:47
Tarazona de Guareña	272	55.9	0:52
Villaflores	260	56.6	0:51
Zorita de la Frontera	152	49.9	0:52
**La Fuente de San Esteban (n = 12); median 61.7 km (range 46.8–70.3)**			
Boada	273	61.2	0:50
Buenamadre	116	62.8	0:50
Cabrillas	324	62.1	0:47
Castraz	37	69.5	0:53
El Cubo de Don Sancho	396	68.3	0:56
Garcirrey	57	46.8	0:45
La Fuente de San Esteban	1,284	56.7	0:43
La Sagrada	84	55.3	0:49
Martín de Yeltes	397	59.4	0:45
Pelarrodríguez	162	67.8	0:55
Retortillo	174	70.3	0:54
San Muñoz	211	47.4	0:40
**Guijuelo (n = 27); median 61.7 km (range 41.7–76.0)**			
Aldeavieja de Tormes	100	52.0	0:42
Berrocal de Salvatierra	72	49.1	0:45
Casafranca	72	60.4	0:54
Cespedosa de Tormes	501	57.8	0:48
El Tejado	91	74.3	1:05

Municipality	Population 2023	Distance (km)	Driving time (h:min)
Fresnedoso	102	65.9	0:51
Fuenterroble de Salvatierra	253	56.5	0:50
Fuentes de Béjar	233	57.2	0:46
Gallegos de Solmirón	109	70.6	1:04
Guijo de Ávila	77	55.3	0:48
Guijuelo	5,484	51.6	0:43
La Cabeza de Béjar	58	58.2	0:46
La Tala	78	63.6	0:58
Ledrada	447	64.8	0:53
Los Santos	592	63.0	0:59
Montejo	211	41.7	0:35
Nava de Béjar	76	59.4	0:46
Navamorales	43	76.0	1:05
Peromingo	122	68.8	0:58
Pizarral	60	44.8	0:38
Puebla de San Medel	33	61.7	0:51
Puente del Congosto	223	71.2	0:59
Salvatierra de Tormes	78	47.2	0:44
Santibáñez de Béjar	452	63.5	0:52
Sorihuela	256	63.2	0:49
Valdelacasa	198	64.5	0:53
Valverde de Valdelacasa	54	67.9	0:56
**Tamames (n = 17); median 66.2 km (range 41.0–82.9)**			
Abusejo	150	66.7	0:52
Alba de Yeltes	201	79.9	1:03
Aldeanueva de la Sierra	106	60.3	1:02
Aldehuela de Yeltes	191	71.1	1:04
Berrocal de Huebra	75	42.2	0:45
Cereceda de la Sierra	66	67.4	1:08
Cilleros de la Bastida	21	66.4	1:12
Dios le Guarde	122	82.9	1:08
La Bastida	28	66.2	1:11
La Rinconada de la Sierra	108	63.2	1:04
Morasverdes	245	79.1	1:16
Narros de Matalayegua	208	41.0	0:45
Navarredonda de la Rinconada	156	57.1	1:04
Puebla de Yeltes	142	64.7	1:08
Sepulcro-Hilario	164	67.1	0:55
Tamames	792	54.1	0:55
Tejeda y Segoyuela	94	61.0	1:01
**La Alberca (n = 9); median 74.2 km (range 66.8–83.6)**			
El Cabaco	238	66.8	1:07
El Maíllo	290	72.2	1:12
La Alberca	1,061	76.5	1:17
Las Casas del Conde	61	76.1	1:17
Mogarraz	253	83.6	1:24
Monforte de la Sierra	59	83.3	1:24
Nava de Francia	122	72.7	1:12
San Martín del Castañar	240	74.2	1:15
San Miguel del Robledo	48	71.3	1:11
**Vitigudino (n = 32); median 75.7 km (range 50.6–92.0)**			
Ahigal de Villarino	31	71.4	1:11

Municipality	Population 2023	Distance (km)	Driving time (h:min)
Almendra	125	72.5	1:13
Barceo	48	75.0	1:15
Bogajo	130	92.0	1:13
Brincones	60	65.7	1:06
Cabeza del Caballo	248	89.0	1:27
Cipérez	242	59.3	0:59
Encinasola de los Comendadores	153	78.2	1:19
Espadaña	34	57.2	0:58
Guadramiro	133	74.4	1:15
Iruelos	26	69.2	1:09
La Peña	72	90.1	1:31
La Vídola	112	86.1	1:26
Moronta	74	86.8	1:12
Peralejos de Abajo	168	63.0	1:03
Peralejos de Arriba	35	61.3	1:02
Pereña de la Ribera	312	90.8	1:31
Pozos de Hinojo	54	79.9	1:06
Puertas	67	63.5	1:04
Sanchón de la Ribera	70	78.0	1:19
Trabanca	175	76.3	1:17
Tremedal de Tormes	37	51.9	0:53
Valderrodrigo	133	78.1	1:18
Valsalabroso	119	82.1	1:22
Villar de Peralonso	209	50.6	0:52
Villar de Samaniego	79	81.4	1:23
Villares de Yeltes	101	72.3	1:07
Villarino de los Aires	775	85.5	1:28
Villarmuerto	39	67.2	1:08
Villavieja de Yeltes	696	85.7	1:07
Vitigudino	2,363	69.2	1:10
Yecla de Yeltes	221	88.8	1:14
**Miranda del Castañar (n = 8); median 81.7 km (range 75.3–106.5)**			
Cepeda	290	82.6	1:27
Garcibuey	200	80.8	1:21
Herguijuela de la Sierra	230	87.3	1:30
Madroñal	140	85.9	1:28
Miranda del Castañar	392	77.8	1:23
Sequeros	215	75.3	1:15
Sotoserrano	525	106.5	1:27
Villanueva del Conde	179	77.6	1:18
**Béjar (n = 24); median 82.7 km (range 65.9–101.9)**			
Aldeacipreste	94	89.2	1:11
Béjar	12,021	73.8	0:59
Candelario	841	76.2	1:02
Cantagallo	275	79.0	1:01
Colmenar de Montemayor	179	96.0	1:17
Cristóbal	166	76.5	1:11
El Cerro	368	89.7	1:10
Horcajo de Montemayor	110	89.7	1:12
La Calzada de Béjar	88	82.4	1:05
La Hoya	31	72.3	0:57
Lagunilla	436	95.0	1:16

Municipality	Population 2023	Distance (km)	Driving time (h:min)
Molinillo	47	82.9	1:17
Montemayor del Río	266	88.7	1:08
Navacarros	132	70.7	0:55
Navalmoral de Béjar	60	78.2	1:00
Peñacaballera	155	84.1	1:04
Pinedas	106	98.4	1:20
Puerto de Béjar	360	82.9	1:03
Sanchotello	222	69.7	0:59
Santibáñez de la Sierra	157	65.9	1:10
Valdefuentes de Sangusín	187	73.9	1:05
Valdehijaderos	83	85.7	1:08
Valdelageve	70	101.9	1:27
Vallejera de Riofrío	67	68.3	0:52
**Aldeadávila de la Ribera (n = 11); median 97.7 km (range 84.9–106.0)**			
Aldeadávila de la Ribera	1,131	103.0	1:41
Barruecopardo	418	93.0	1:31
Cerezal de Peñahorcada	73	92.9	1:31
El Milano	93	86.3	1:25
La Zarza de Pumareda	138	96.5	1:34
Masueco	262	102.3	1:44
Mieza	179	97.7	1:37
Saldeana	105	99.7	1:39
Saucelle	254	101.8	1:40
Villasbuenas	168	84.9	1:25
Vilvestre	394	106.0	1:49
**Ciudad Rodrigo (n = 14); median 101.8 km (range 71.3–117.6)**			
Carpio de Azaba	111	100.3	1:10
Castillejo de Martín Viejo	219	104.6	1:20
Ciudad Rodrigo	11,810	89.3	1:04
El Bodón	245	103.7	1:18
La Atalaya	100	105.6	1:22
La Encina	101	102.0	1:17
Monsagro	133	117.6	1:31
Pastores	54	101.1	1:17
Saelices el Chico	173	101.1	1:16
Sancti-Spíritus	738	71.3	0:52
Serradilla del Arroyo	248	106.9	1:22
Serradilla del Llano	145	109.0	1:23
Tenebrón	133	101.7	1:13
Zamarra	75	101.4	1:18
**Fuentes de Oñoro (n = 10); median 115.2 km (range 107.8–143.0)**			
Aldea del Obispo	275	127.3	1:29
Espeja	211	107.8	1:15
Fuentes de Oñoro	1,043	114.9	1:20
Gallegos de Argañán	269	109.8	1:17
La Alameda de Gardón	79	114.9	1:23
La Bouza	47	143.0	1:43
Puerto Seguro	68	134.3	1:40
Villar de Argañán	98	115.5	1:22
Villar de Ciervo	266	123.5	1:30
Villar de la Yegua	159	112.0	1:27

Municipality	Population 2023	Distance (km)	Driving time (h:min)
**Robleda (n = 8); median 116.6 km (range 108.9–133.2)**			
Agallas	126	117.1	1:33
El Payo	325	133.2	1:44
El Sahugo	177	114.5	1:29
Herguijuela de Ciudad Rodrigo	70	108.9	1:24
Martiago	264	112.1	1:27
Peñaparda	319	127.6	1:40
Robleda	462	116.1	1:29
Villasrubias	245	121.9	1:34
**Lumbrales (n = 12); median 118.1 km (range 83.6–131.0)**			
Ahigal de los Aceiteros	94	119.8	1:39
Bañobárez	280	102.6	1:23
Bermellar	131	125.1	1:43
Cerralbo	119	83.6	1:23
Fuenteliante	89	97.7	1:18
Hinojosa de Duero	600	126.4	1:44
La Fregeneda	310	131.0	1:48
La Redonda	64	120.2	1:39
Lumbrales	1,517	116.4	1:35
Olmedo de Camaces	100	108.0	1:28
San Felices de los Gallegos	354	112.0	1:31
Sobradillo	182	123.4	1:42
**Fuenteguinaldo (n = 8); median 125.2 km (range 114.7–135.5)**			
Campillo de Azaba	141	116.3	1:22
Casillas de Flores	179	124.0	1:36
Fuenteguinaldo	662	114.7	1:28
Ituero de Azaba	205	120.2	1:25
La Alamedilla	108	135.2	1:39
La Alberguería de Argañán	103	133.8	1:38
Navasfrías	370	135.5	1:46
Puebla de Azaba	145	126.4	1:31
**Urban zones of Salamanca city (n = 11)**			
San Bernardo Oeste	§	1.7	0:05
Tejares	§	1.8	0:06
Pizarrales	§	2.4	0:07
Centro-Universidad	§	2.5	0:06
San José	§	2.5	0:06
Sancti-Spiritus-Canalejas	§	2.5	0:08
San Juan	§	2.9	0:08
Capuchinos	§	3.1	0:08
Garrido Sur	§	3.2	0:10
La Alamedilla	§	3.2	0:10
Garrido Norte	§	3.3	0:10

Note: Distances and driving times are road estimates (fastest route, no traffic) calculated with OSRM on OpenStreetMap road data from the main settlement of each municipality to the University Hospital of Salamanca (reference hospital); municipalities were geocoded with Nominatim. Population: INE official figures, 2023. Municipalities are listed under the primary healthcare zone to which they belong. § Population of the municipality of Salamanca (143,954 in 2023). Distances are municipality-level estimates and do not reflect the residence or travel of individual patients.
